# Malaria control – lessons learned from trends of Malaria indices over three decades in Karnataka, India

**DOI:** 10.1186/s12936-023-04774-1

**Published:** 2023-11-17

**Authors:** Vani Hanumantappa Chalageri, Shrinivasa B. Marinaik, Sujith N. Nath, Richa Singhal, Smita Rawat, Krishnappa Ravikumar, Mahamood Shariff, Alex Eapen

**Affiliations:** 1 ICMR - National Institute of Malaria Research Field Unit, Bengaluru, 562110 Karnataka India; 2 ICMR - National Institute of Malaria Research, New Delhi, India; 3 Regional Office for Health & Family Welfare (ROHFW), Chief Medical Officer, Bengaluru, Karnataka India; 4 Regional Office for Health & Family Welfare (ROHFW), Ex-Senior Regional Director, Bengaluru, Karnataka India; 5 Deputy Director, NCVBDC, Directorate of Health & FW Services, Bangalore, Karnataka India; 6 ICMR- National Institute of Malaria Research Field Unit, Chennai, 600077 Tamil Nadu India

**Keywords:** Malaria, Decadal trends, Malaria elimination, *P. Falciparum*, Annual Parasite Index

## Abstract

**Background:**

Karnataka is one of the largest states in India and has a wide range of geographical terrains, ecotypes, and prevalence of malaria. It experiences a voluminous influx and efflux of people across the state that affects the spread of malaria. The state deployed focused intervention measures keeping the national objective of malaria elimination as the foremost priority. This brought down malaria cases below a thousand by the year 2021. Furthermore, the state is motivated toward malaria elimination by 2025. This study analyzes the trends in malaria indices over the past three decades in the state and highlights the key intervention measures that impacted the reduction in the malaria burden.

**Methods:**

Data from 1991 to 2021 at the district level was collected from the archives of Regional Office for Health & Family Welfare (ROH&FW), Bangalore. Time-tend analysis on this data was conducted after categorization into three decades. Sequence plots were then plotted on the moving average of Annual Parasite Index for all those three decades. Generalized estimating equation model with Poisson distribution were used to evaluate difference in these indicators with pre and post interventions like LLIN, RDT with ACT and Guppy and Gambusia fishes.

**Results:**

Malaria burden across the state has consistently declined over the last three decades with few years of exception. This has coincided with the mortality also steadily declining from 2006 and culminating in zero malaria deaths reported from 2011 to 2019. Morbidity had drastically reduced from the hundred-thousand (1993–2003) to ten thousand (2004–2016) thousands (2017–2020) of cases in this period and less than thousand cases were reported by 2021. Generalized estimating equation (GEE) model revealed significant difference of incidence risk ratio of malaria incidence and deaths, post introduction of interventions like LLIN, RDT with ACT and Guppy and Gambusia fishes, indicating these three as important interventions for reducing the malaria burden. Time trend analysis revealed a linear decreasing trend in malaria cases during 2011–2021 decade.

**Conclusions:**

A linear decreasing trend in malaria cases was observed during 2011–2021 decade. LLIN, RDT with ACT and Guppy and Gambusia fish’s interventions significantly helped in reducing the state malaria burden.

## Background

The 2022 World Malaria Report highlights India’s contribution to malaria burden in the South-East Asia Region [1], with major circulating parasites being *Plasmodium falciparum* and *Plasmodium vivax.* According to the report, India accounted for 79% and 83% of the region’s morbidity and mortality, respectively.

The prevalence of malaria in India is heterogeneous, with the highest numbers among tribal population and forest dwellers [2–4]. The varied landscapes and ecosystems coupled with constantly fluctuating climatic conditions, have a significant impact on breeding and survival of *Anopheles* mosquitoes, which serve as the primary carriers of malaria in India [5, 6]. Other challenges include continuous migration, undetected malaria cases, symptomatic/sub-patent malaria cases, multiple vectors, treatment failure, resistant malaria and insecticide resistance among mosquitoes. The shortage of skilled manpower, lack of reporting from the private health care providers, and unplanned expansion of urban and semi-urban areas widens the concern [5]. India launched the National Framework for Malaria Elimination in 2016, aimed at eliminating malaria by 2030. The programme outlines strategies and guidelines for achieving this target in a time-bound manner. Through its focused implementation, significant reduction in the country’s malaria mortality and morbidity was witnessed by 2020.

Karnataka is located within the latitudinal range of 11.5° North to 18.5° North and the longitudinal range of 74° East to 78.5° East [7]. covering an area of 191,791 square km, thereby being the seventh largest state in India [8]. Karnataka displays diverse geographical terrains, from coastal line to tropical forests of Western Ghats and Deccan plateau. Agriculture and livestock remain the predominant occupation of the state [9]. Human - animal cohabitation, which is an inherent part of the country’s agrarian system also contribute to the propagation and survival of mosquito vectors. Sporadic malaria outbreaks are regularly reported in the Western Ghats, coastal regions (both receiving heavy rainfall), and also in the arid (very dry) northern districts [10]. The dependency of people on wells [10, 11], and reluctance in using mosquito nets [12] serve as attenuating factors for mosquito breeding and biting, respectively. Karnataka state has set itself the goal of malaria elimination by 2025 [10]. The adherence to and implementation of anti-malarial strategies would be key to achieving elimination, five years before the national target.

This study analyses trends in malaria indices over the past three decades in Karnataka and highlights key intervention measures that impacted reduction in the malaria burden.

## Methods

The state health department of Karnataka mandates reporting and surveillance of all malaria cases to the national programme through a series of forms (M1, M2, M3, M4) that are used by various healthcare providers/facilities to report cases and request slide examinations [10]. The data collected through these forms are an integral part of the reporting system at primary health centres, and has been functioning long before the start of data collection by NVBDCP (National Vector Borne Disease Control Programme) (2003) and IDSP (Integrated Disease Surveillance Programme). The Regional Office for Health & Family Welfare (ROH&FW), Bangalore, has archived all these reports and this data from 1991 to 2021 was retrieved for analysis. The indices, percentage of *P. vivax* and *P. falciparum* were all calculated as per guidelines [13]. The absolute number of cases, deaths and blood slides examined are illustrated as graphs in the manuscript.

**Table:** 

Indicator	Numerator	Denominator	Multiplying factor
Annual Parasite Index (API)	Total number of blood smears positive for malaria parasite in a year	Total population	1000
Annual Blood Examination Rate (ABER)	Number of blood smears examined in a year	Total population	100
Slide Positivity Rate (SPR)	Total number of blood smears found positive for malaria parasite	Total number of blood smears examined	100
Slide falciparum rate (SFR)	Total number of blood smears found positive for *P. falciparum*	Total number of blood smears examined	100
*P. falciparum* percentage (Pf %)	Total number of blood smears found positive for *P. falciparum*	Total number of blood smears positive for malaria parasite	100
*P. vivax* percentage (Pv %)	Total number of blood smears found positive for *P. vivax*	Total number of blood smears positive for malaria parasite	100

For the years 2004-05 later when RDT was introduced, numerator and denominator included values of blood smears and RDTs. The values include both active and passive surveillance data

Overall, it may be understood that the methods used for malaria reporting and surveillance in Karnataka involved a well-established system of forms and data collection. The use of various rates (ABER, API, SPR, SFR) facilitates a comprehensive understanding of the malaria situation in the state.

Trends in malaria indicators during the three decades under study were assessed by computing two years moving average. To conduct time-tend analysis, data was categorized into three decades: (1) years 1991–2000, (2) years 2001–2010 1), years 2011–2021. Two years moving average was calculated for smoothening the data curves by eliminating random numbers which may arise due to sudden increase or decrease in cases during a particular year. Sequence plots were plotted on the moving average of Annual Parasite Index (API) for all three decades. API has been calculated as the number of malaria positive cases per 1000 populations in a particular year. Another indicator studied is the death rate per 1,00,000 populations in a particular year.

Trends in API and death rate before and after the introduction of various community level interventions, including the distribution of Long-lasting insecticidal nets (LLIN), the case management using Rapid diagnostic tests (RDT) and artemisinin-based combination therapy (ACT), biological vector control using the release of Guppy and Gambusia fishes, were assessed. Generalized estimating equation (GEE) model with Poisson distribution were used to evaluate difference in these indicators before and after the introduction of these interventions. A predictor variable was created to categorize the year wise data into binary form. Categorization based on the year, was done based on the year of introduction of a particular community level intervention. To assess the trends after introduction of LLIN in 2009, data was categorized year-wise, where 1991–2009 was treated as before intervention and 2010–2021 as after intervention period. To assess the effect of the introduction of ACT and RDT, years from 1991 to 2005 were categorized as before intervention and years from 2006 to 2021 as after intervention. Years for assessing the effect of Guppy and Gambusia fishes were stratified as 1991–1996 pre-intervention and from 1997 to 2021 as post-intervention.

To evaluate the temporal trends pre- and post-intervention of various community level programmes, GEE models were created by stratifying data on time periods of various interventions and taking year as a predictor variable. Incidence Rate Ratio (IRR) with 95% confidence interval have been reported. All the analysis was done using SPSS version 29.0 and a p-value of < 0.05 was considered as significant.

## Results

**Fig. 1:**
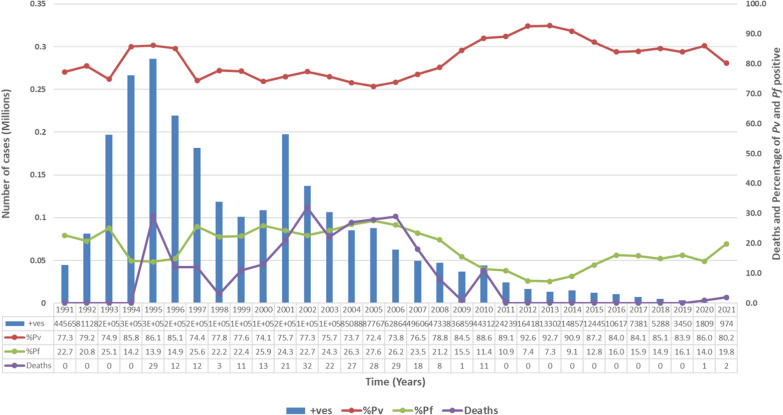
Mortality and morbidity trends of malaria from 1991-2021 in Karnataka. +ves- total number of positive malaria cases detected through smear/RDT. %Pv – percentage of P vivax cases detected during that year. %Pf- percentage of P falciparum cases detected during that year

 There has been a significant decline in malaria morbidity and mortality in Karnataka over the past three decades. Few spikes in malaria mortality in Karnataka were observed during 1995 (29 deaths), 2002 (32 deaths) and 2006 (29 deaths) (Fig. 1). Zero malaria deaths were reported in the state from 2011 to 2019, indicating its success implementation of the malaria control programme. The incidence rate of *P. vivax* between 1991 and 2007 was 77.53 (SD = 4.29) compared to an incidence rate of *P. falciparum* of 22.47 (SD = 4.29) (P < 0.01). However, from 2008 to 2021, the incidence rate *P. vivax* was 84.93 (SD = 5.72) compared to the incidence rate of *P. falciparum* of 13.43 (SD = 4.22) (P < 0.01), indicating a higher burden of *P. vivax* cases.

**Fig. 2:**
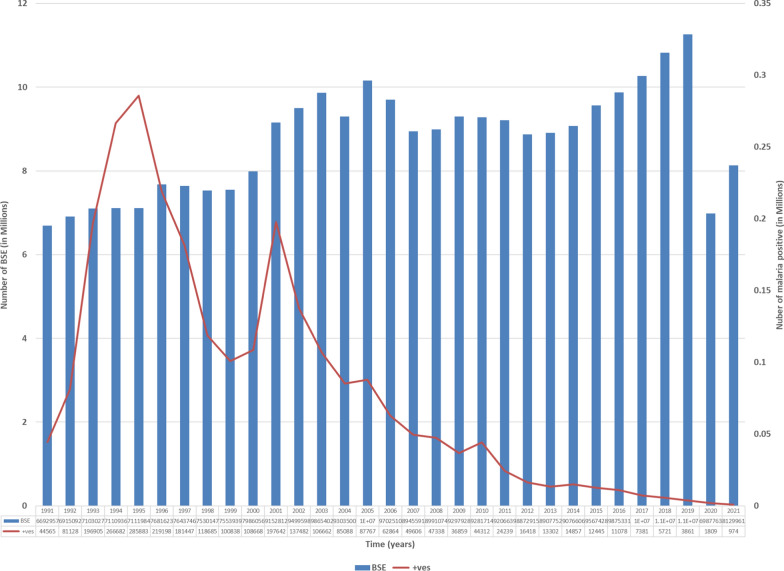
Trends of malaria surveillance in Karnataka (1991-2021). *BSE* Blood smear examined. +ves- total number of positive malaria cases detected through smear/RDT

 Figure 2 describes the adequacy of testing for malaria diagnosis when using active and passive surveillance data. The graph indicates that there has been a consistent increase in the number of Blood smear examination count (BSE) from 1991 to 2021, with a range of 6.5 million to 11.2 million tests being conducted annually, except for a dip in the year 2020 (6,987,763 cases tested and 1809 identified as malaria positive). Even with adequate tests being carried out, the number of malaria positive cases has drastically declined in this period, except in the years 1995 (285,883 positive cases) and 2001 (197,642 positive cases). Reported malaria cases in Karnataka reduced below 1000 for the first time in 2021 (974). Moreover, the number of tests conducted in 2021 (8.1 million - ABER of 13.75%) is more than the ABER target of 10% set by the national programme.

**Fig. 3:**
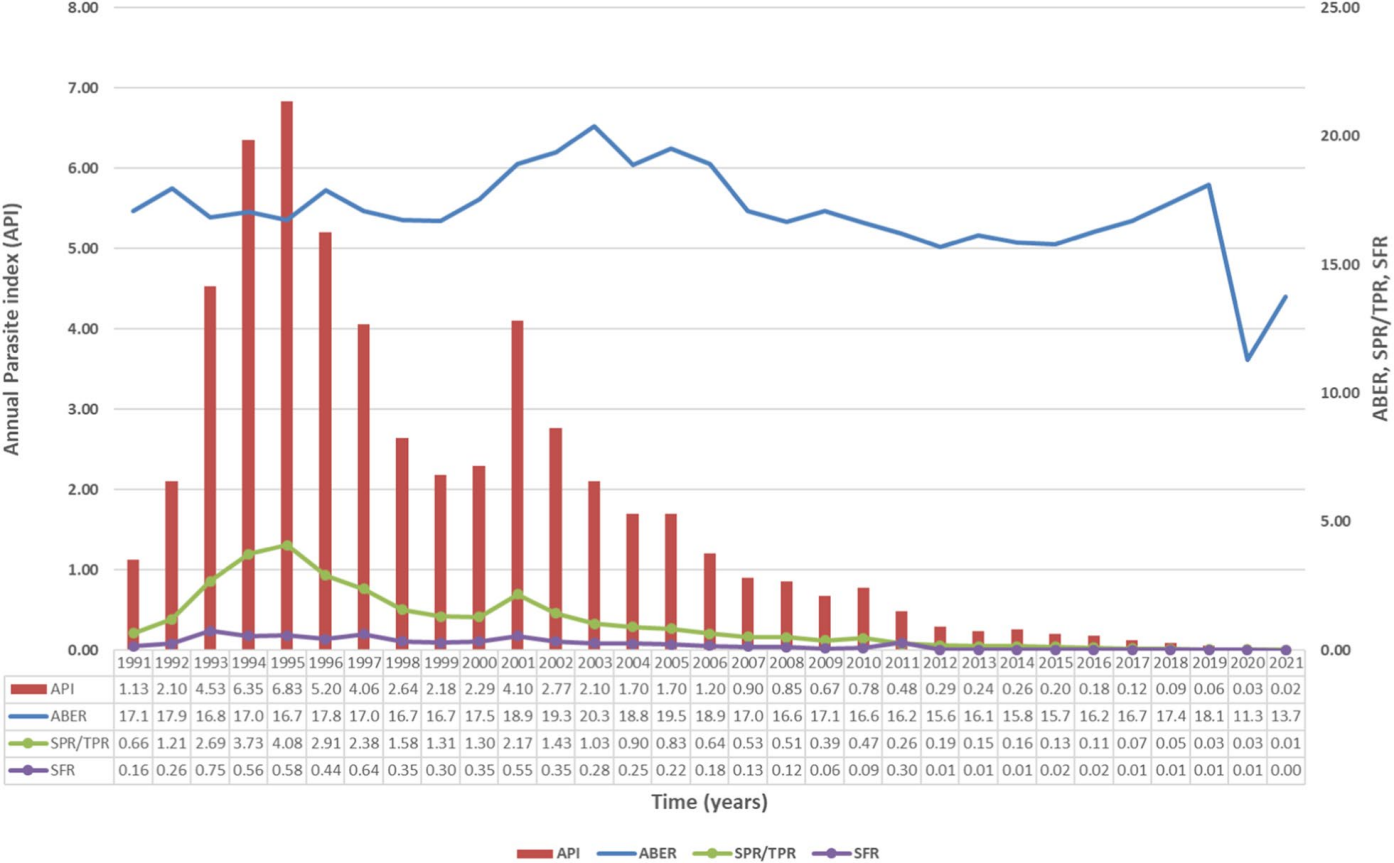
Key malariometric indices of malaria epidemiology in Karnataka (1991-2021). (*API*) Annual Parasite Index, (*ABER*) Annual Blood Examination Rate, (*SPR*) slide positivity rate or (*TPR*) Test Positivity rate, (*SFR*) slide falciparum rate

 It can be observed (Fig. 3) that, the API had been above 2 in the first quarter of this period and reached its peaks in the years, 1993–1997 and in 2001. This correlates with the increase in malaria cases during those years. The API was consistently below two since 2004 and further declined to less than one in the period from 2007 to 2021. The ABER had been consistently maintained above 15% during these three decades, (much greater than the national ABER target of 10%), indicating the quality of the state surveillance system. The ABER had dropped to 11.3 in 2020, which may be due to the impact of the COVID-19 pandemic, but the API (0.03) continued to remain adequate even 2021. SPR had peaked during the years 1994 (3.73) 1995 (4.08) and 2001 (2.17) when cases were also at its peak. However, the SPR has been consistently less than one since 2002 even with an adequate percentage of ABER.

### Time trend analysis of the three decades

Analysis of data on various malaria indicators of the state for three decades (1991–2021) revealed that malaria progressed exponentially during the first decade (1991–2000), as evident from the annual parasite incidence per 1000 population and the death rate per 1,00,000 population. The next two decades (2001–2010 and 2011–2021) witnessed a sharp decline in cases, which may be attributed to effective malaria control strategies at the community level and other measures adopted as a part of malaria elimination programme.

**Fig. 4:**
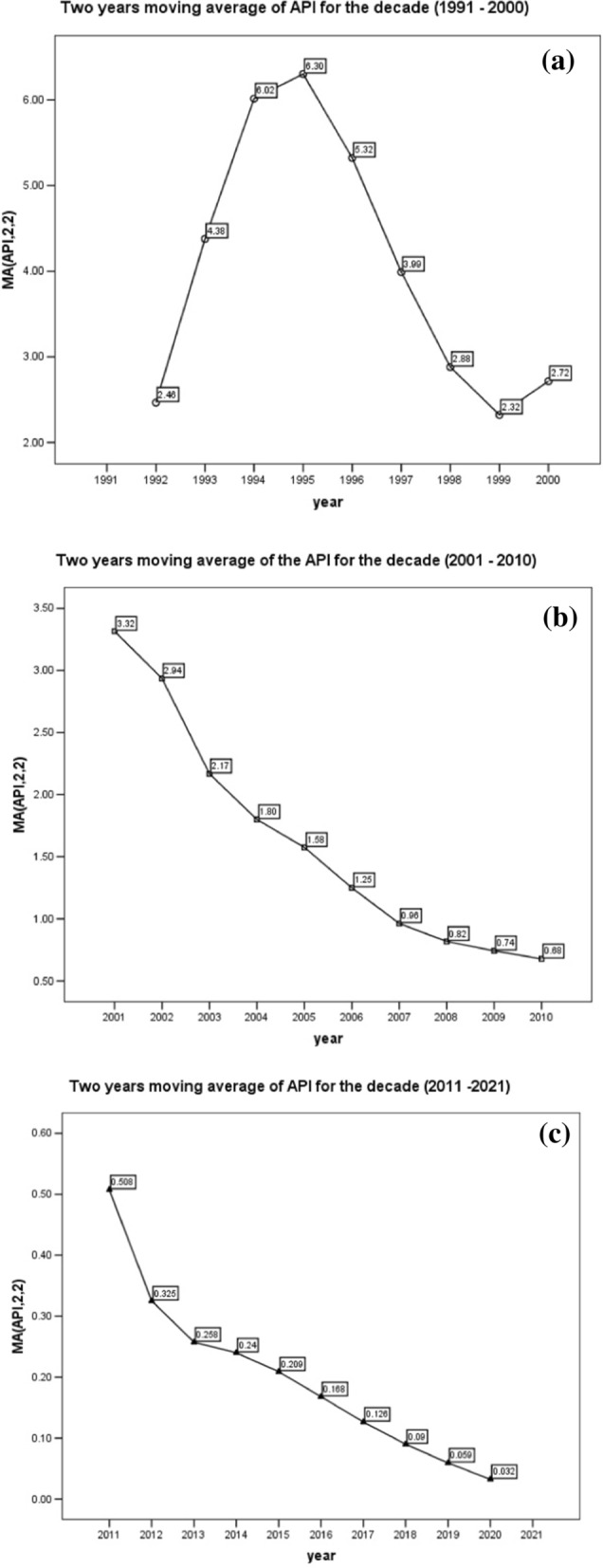
**a** Time trend analysis for the decade 1991–2000. **b** Time trend analysis for the decade 2001–2010. **c** Time trend analysis for the decade 2011–2021

 Karnataka witnessed a steep increase in malaria incidence rate between 1991 and 2000. Confirmed malaria cases increased from 44,565 to 1991 to 1,08,668 in 2000, an estimated average increase of 26.6% per year. The API raised from 1.13 to 2.29 per 1000/population. Malaria confirmation rate also increased from 0.66 to 1.3%, as evident from the Slide positivity rate. The malaria death rate also increased from 0 to 0.029 per 1,00,000 population. The two years moving average of API revealed that malaria cases were on the rise from the year 1991 till 1995, after which a linear decline in the trends was observed till 2000 (Fig. 4a).

The introduction of major community level interventions for malaria control resulted in a strident drop of malaria incidence and death rates during the next two decades. The confirmed malaria cases decreased from 1,97,642 to 44,312, during the next decade (2001–2010), showing an average decrease of 17.1% per year. API decreased from 4.1 to 2001 to 0.78 in 2010 per 1000 population. Malaria death rate also witnessed a decline from 0.045 to 0.020% per 1,00,000 population. An exponential decreasing trend in malaria cases was observed during this decade as evident from the two years moving average trend, which may be attributed to major malaria control interventions undertaken during this decade, such as the introduction of ACT, RDT and LLIN (Fig. 4b).

In the subsequent decade (2011–2021) further decrease in confirmed malaria incidence rate was observed. The confirmed malaria cases were found to be 24,239 in 2011 which decreased to 974 in 2021 (an average decrease of 25.4% per year). API during this decade dropped from 0.48 to 0.015 per 1000 population. This decade also witnessed “zero” malaria deaths from 2011 to 2019, while only 1 and 2 deaths due to malaria were observed in years 2020 and 2021 respectively. Time trend analysis revealed a linear decreasing trend in malaria cases during this decade (Fig. 4c).

The comparison of various indicators before and after the introduction of LLIN in the year 2009 revealed that there was a decrease of 89.7% (IRR = 0.103, P < 0.001) in the malaria incidence rate after LLIN expansion (2010–2021) as compared to before its expansion (1991–2009). The death rate also decreased by 91.7% (IRR = 0.083, P = 0.001) after LLIN introduction in 2009. A decrease of 85.1% (IRR = 0.149, P < 0.001) was observed in the malaria incidence after introduction of ACT and RDT (2006–2021) as compared to before its introduction (1991–2005). The death rate also decreased by 68.8% (IRR = 0.312, P = 0.022) after expansion of ACT and RDT.

After the introduction of Guppy and Gambusia fishes as biological vector control there was a decrease of 67.6% in malaria incidence rate compared to pre-introduction (IRR 0.324, P < 0.001). However, there was slight increase in death rate (IRR = 1.399, P = 0.625), post 1995 (Table 1).

**Table 1:** Comparison of the period before and after various interventions

Before and after intervention as binary predictor variable		Malaria incidence	Death incidence
LLIN introduction ^a^	IRR (95% CI)	0.103 (0.059, 0.180)	0.083 (0.018, 0.377)
	p-value	< 0.001 (*)	0.001 (*)
ACT and RDT introduction ^b^	IRR (95% CI)	0.149 (0.092, 0.243)	0.312 (0.115, 0.846)
	p-value	< 0.001 (*)	0.022 (*)
Introduction of Guppy and Gambusia fishes as biological vector control ^c^	IRR (95% CI)	0.324 (0.188, 0.558)	1.399 (0.364, 5.373)
	p-value	< 0.001 (*)	0.625

*IRR* Incidence rate ratio, *LLIN* Long lasting insecticidal treated nets, *ACT* Artemisinin-based combination therapies, *RDT* Rapid Diagnostic Testing

(*) denotes p-value is significant at 5% level of significance

^a^ Years from 1991–2009 considered as before LLIN introduction and 2010–2021 as after LLIN introduction

^b^ Years from 1991–2005 considered as before ACT and RDT introduction and 2006–2021 as after ACT and RDT introduction

^c^ Years from 1991–1996 considered as before introduction of Guppy and Gambusis fishes and 1997–2021 as after introduction

### Comparison of the effect of community interventions on Malaria indicators during different time periods

The effect of various community level malaria control interventions was also assessed separately for time periods before and after their introduction. Malaria incidence declined by 22.5% (IRR = 0.775, P < 0.001) during the period (2010–2021) after LLIN introduction in year 2009. Death rate was 107% times higher (IRR = 1.070, P = 0.076) before its introduction which decreased by 28.6% (IRR = 0.714, P = 0.241) each year after LLIN introduction (Table 2). The introduction of ACT and RDT during 2004–2005 significantly impacted the death rate as well as malaria incidence. Overall malaria incidence rate decreased by 18.6% every year (IRR = 0.814, P < 0.001) during the period (2006–2021). Death rate was on rise 117% (IRR = 1.173, P = 0.002), before the introduction of ACT and RDT during the period (1991–2005) which decreased by 35.7% after its introduction (IRR = 0.643, P < 0.001) (Table 2).

**Table 2:** Average annual change in malaria and death incidences before and after introduction of various interventions

Intervention: Predictor variable: Year	Period	Malaria incidence		Death incidence	
		IRR (95% CI)	*p-value*	IRR (95% CI)	*p-value*
LLIN	1991–2009	0.942 (0.900, 0.987)	0.012	1.070 (0.993, 1.153)	0.076
	2010–2021	0.775 (0.739, 0.812)	< 0.001 (*)	0.714 (0.407, 1.253)	0.241
ACT and RDT	1991–2005	0.971 (0.917, 1.029)	0.321	1.173 (1.061, 1.297)	0.002 (*)
	2006–2021	0.814 (0.798, 0.830)	< 0.001 (*)	0.643 (0.519, 0.795)	< 0.001 (*)
Guppy and Gambusia fishes as biological vector control	1991–1996	1.288 (1.091, 1.522)	0.003 (*)	2.345 (1.347,4.081)	0.003 (*)
	1997–2021	0.867 (0.850, 0.885)	< 0.001 (*)))	0.892 (0.848, 0.938)	< 0.001 (*)

*IRR* Incidence Rate Ratio

(*) denotes p-value is significant at 5% level of significance

Biological vector control measures, such as the introduction of Guppy and Gambusia fishes, also aided in reducing malaria incidence by 13.3% (IRR = 0.867, P < 0.001) during the year 1997. Reported number of deaths had increased during the years 1995 and 1996 and introduction of biological control measures along with other community interventions aided in reducing the death rate by 10.8% (IRR = 0.892, P < 0.001) during the period (1997–2021) (Table 2).

## Discussion

Karnataka in the past, faced a significant malaria burden affecting both rural and urban areas. To address this issue, the state implemented site-specific interventions of anti-malarial units in 1991, under the Krishna Bhagya Jala Nigam Limited (KBJNL) programme. These units were responsible for conducting vector and disease control activities in respective areas. Out of the various cities in the state [14], Mangalore has been reporting the highest number of cases over the past decades [10, 15] and even in 2021, Mangaluru, Udupi and rural UPK reported the highest number of cases.

Karnataka had adopted the objectives and goals of the National Malaria Control Programme to its health system. The programme evolved into National Vector Borne Diseases Control Programme in 2003 and currently comes under the administration of the National Centre for Vector Borne Diseases Control. The adoption of Integrated Vector Management programme, which included the use of the insecticide malathion, and synthetic pyrethroids in 1996, was another step in the right direction. Karnataka accelerated the pace of malaria elimination by focusing heavily on the spots and selected endemic zones using intervention measures, such as the introduction of ACT (2004–2005), of RDTs (2004–2005), of LLINs (2009) and the incorporation of the revised National Malaria Drug Policy (2013) [16]. Malaria was made a notified disease under the Epidemic Disease Act of 1987 [10] in 1998 and it probably resulted in increased reporting of cases. The introduction of Guppy and Gambusia fishes as biological vector control measures were undertaken from 1996 to 2000^9^. GEE analysis show significant reduction in malaria morbidity (IRR 0.324, P < 0.001) post-introduction. Health education programmes have also been identified as effective by certain studies [17, 18]. A ban on oral artemisinin monotherapy was also imposed in this period to prevent the emergence of artemisinin-resistant malaria parasites, which is a major concern in the Southeast Asia region. Karnataka state with high number of public health institutions and trained health workers [19] is presently able to effectively implement early diagnosis and treatment, which is a prerequisite for malaria control.

Furthermore, the state has taken proactive measures like; employing district level entomologists, constituting rapid response teams for outbreak investigation and extending additional support in terms of manpower, capacity building and logistics to malaria endemic areas [10]. It can be deduced that the cumulative effect of multiple interventions is the reason for the significant decline of malaria cases in Karnataka. The state has been able to sustain nil indigenous cases of malaria in most districts, which is a significant milestone towards malaria elimination.

The COVID-19 pandemic had posed significant challenges to malaria control programmes worldwide. The overlapping symptom of fever for malaria and COVID-19 might have resulted in misdiagnosis of malaria cases, and could have also led to co-infections being overlooked in non-endemic regions [20]. The diversion of funds and manpower from malaria control to COVID-19 control may have also affected vector control measures, malaria diagnosis, treatment, and surveillance activities, leading to a low number of reported malaria cases [21]. Moreover, the usage of hydroxychloroquine as chemoprophylaxis for COVID-19 disease, might have also masked the symptoms of malaria patients [21]. Interruptions of services across the world were seen due to COVID-19 [22], but despite these challenges, the malariometric indices observed in Karnataka were within the state control. There were certain limitations in the monthly report data archived at the Regional Office for Health & Family Welfare (Bangalore) and the breakdown of malaria cases by age and gender were not available. This prevented the study from analysing cohort level impact and gender variances in effectiveness of the intervention measures.

### Strengths of the state

Among the various interventions implemented for state malaria control, the introduction of RDT and ACT produced a successful decline in malaria morbidity and mortality. With LLIN and Guppy & Gambusia fish introduction, a significant decline in malaria morbidity was observed in the state. The implementation of these three successful interventions in Karnataka, can be considered a role model for rest of the country. As a result of its performance in the field of vector borne disease control, on the occasion of World Malaria Day in 2022, Karnataka was praised as one of the best performing states in the country [23]. The state was also elevated from category 2 to category 1 (states/UTs with API < 1, and all the districts in the state with API < 1) in terms of malaria elimination [24]. Karnataka’s success highlight the importance of well-coordinated and sustained effort towards malaria control, which involves multiple stakeholders such as the government, healthcare providers, and the community [25–27].

## Conclusion

Data trends from the past three decades reveal a reduction in malaria burden across the state. This was made possible through adequate and sustained surveillance activities. Successfully proven interventions such as LLIN, Guppy and Gambusia fishes, RDT and ACT, can be implemented in other regions with similar eco-epidemiological settings. Karnataka has set itself the target of achieving malaria elimination by 2025 and being in the pre-elimination stage, it is crucial to conduct further research to assess and review the current malaria control and elimination strategies. By doing so, Karnataka can lead the nation towards malaria elimination, by setting an example for others to follow.
